# Breeding for plant heat tolerance at vegetative and reproductive stages

**DOI:** 10.1007/s00497-016-0275-9

**Published:** 2016-02-13

**Authors:** Nicky Driedonks, Ivo Rieu, Wim H. Vriezen

**Affiliations:** Department of Molecular Plant Physiology, Institute for Water and Wetland Research, Radboud University, Heyendaalseweg 135, 6525 AJ Nijmegen, The Netherlands; Bayer CropScience Vegetable Seeds, PO Box 4005, 6080 AA Haelen, The Netherlands

**Keywords:** Heat tolerance, Plant breeding, Plant biotechnology, Crops, Knowledge transfer

## Abstract

*****Key message***:**

**Thermotolerant crop research.**

**Abstract:**

Global warming has become a serious worldwide threat. High temperature is a major environmental factor limiting crop productivity. Current adaptations to high temperature via alterations to technical and management systems are insufficient to sustain yield. For this reason, breeding for heat-tolerant crops is in high demand. This review provides an overview of the effects of high temperature on plant physiology, fertility and crop yield and discusses the strategies for breeding heat-tolerant cultivars. Generating thermotolerant crops seems to be a challenging task as heat sensitivity is highly variable across developmental stages and processes. In response to heat, plants trigger a cascade of events, switching on numerous genes. Although breeding has made substantial advances in developing heat-tolerant lines, the genetic basis and diversity of heat tolerance in plants remain largely unknown. The development of new varieties is expensive and time-consuming, and knowledge of heat tolerance mechanisms would aid the design of strategies to screen germplasm for heat tolerance traits. However, gains in heat tolerance are limited by the often narrow genetic diversity. Exploration and use of wild relatives and landraces in breeding can increase useful genetic diversity in current crops. Due to the complex nature of plant heat tolerance and its immediate global concern, it is essential to face this breeding challenge in a multidisciplinary holistic approach involving governmental agencies, private companies and academic institutions.

## Introduction

Ambient temperatures are rising at a considerable rate as part of the current global climate change. The last three decades are thought to be the warmest the earth has experienced in the past 1400 years in the Northern Hemisphere. Climate models predict that the global mean temperature will continue this trend, increasing by 1–4 °C by the end of the twenty-first century. Additionally, climatological extremes such as heat waves are likely to occur more frequently (IPCC 2013; Tebaldi et al. 2006; Hansen et al. 2015). While the above data refer to average global temperature increases, there are significant regional and seasonal differences with further potential impact on agriculture (IPCC 2007). The biggest temperature changes will be at higher latitudes (IPCC 2007). In these regions, the increase in temperature might benefit overall crop production by alleviating low-temperature growth inhibition at the start of the growing season, allowing earlier planting of crops, and the possibility of a longer growing season or more cropping cycles per year in the longer term (Gitay et al. 2001). Thus, a rise in temperature is expected to lead to expansion of areas suitable for crop production in the Russian Federation, North America and Northern Europe as well as in East Asia (Lotze-Campen and Schellnhuber 2009; Olesen and Bindi 2002). Offsetting these benefits, however, are negative effects at lower latitudes, where temperatures are already at the higher end of the crops’ optimal grow temperature ranges. Regions in Africa, for example, have been predicted to become (semi-)arid due to heat and water stress, resulting in significant yield losses (Fischer et al. 2002; Ortiz et al. 2008), and in Asia and the Middle East, crop yields are predicted to fall 15–35 % if the average temperature increases 3–4 °C (Ortiz et al. 2008; FAO 2009).

More than 200,000 plant species are estimated to exist globally, of which ~80,000 are edible to humans. Despite this huge variety, 95 % of the calories and protein intake of human and livestock are derived from only 20–25 species (Füleky 2009). For example, only three species, wheat, rice and maize, account for 75 % of global grain production (Bansal et al. 2014; Lobell and Gourdji 2012). Breeding and agronomic improvements of these species have resulted in increased production between 1985 and 2005, and previous IPCC projections assumed that this will continue in the future (Ainsworth and Ort 2010; Teixeira et al. 2013). However, based on an extended update of the IPPC projections, a new meta-study predicted a less optimistic scenario. Primarily due to a more negative effect of moderate warming on yield, worldwide yield reductions are now expected for wheat, rice and maize in both tropical and temperate regions under a scenario of 2 °C of local warming without adaptation (Challinor et al. 2014). Relative rates of yield increase for major cereal crops are already declining (Fischer and Edmeades 2010; Foley et al. 2011). However, to meet the demand for food from the population of an expected 9 billion people in 2050, a 70 % increase in food production is deemed necessary according to the Declaration of the World Summit on Food Security (FAO 2009). This means that the yearly increases in production for the coming 40 years need to be 38 % higher than those achieved historically (Tester and Langridge 2010).

Thus, the temperature changes associated with global warming have become a major challenge with respect to agricultural output. Here, we provide an overview of research that aims to support increasing heat tolerance of crops. We evaluate potential changes to crop management, the availability of genetic resources for breeding activities and the usefulness of (academic) research into the molecular and physiological basis of heat tolerance. Finally, it is discussed whether academic research and breeding activities are complementary with mutual benefits.

## Effects of high temperature on crops

### General physiological effects of high temperature

The intensity, duration and rate of temperature change together determine the impact of high temperature on plant development and physiology (Wahid et al. 2007; Zinn et al. 2010). Air and soil temperature affect crop yield in different ways, and this should be considered when studying heat impact (Lobell and Gourdji 2012; Sharkey and Schrader 2006). Plant physiological responses to heat stress have been reviewed in great detail (Wahid et al. 2007; Bita and Gerats 2013; Bokszczanin et al. 2013; Mathur et al. 2014). In general, a moderate increase in air temperature leads to faster plant development and a shorter crop duration and consequently a reduction in cumulative light perception and assimilation over the plant’s life cycle. In addition, disturbance of fundamental processes such as carbon assimilation, respiration and transpiration may reduce overall metabolic efficiency and result in vegetative developmental defects such as fewer, malformed and/or smaller organs (Takeoka et al. 1991; Maestri et al. 2002; Stone 2001). High air temperature can also negatively affect sexual reproduction and consequently fruit and seed yield (Peet et al. 1997; Erickson and Markhart 2002; Zinn et al. 2010). On the other hand, high soil temperature can reduce germination capability and plant emergence and can cause heat necrosis of roots (Stevenson et al. 2001).

### Heat sensitivity in crop plants

Individual plant yield is a function of various components including plant architecture, photosynthetic efficiency, resource partitioning and reproductive success—each of these components may be vulnerable to heat. Optimum temperature range and consequently heat sensitivity vary among crop types, species and cultivars (Ulukan 2008; Levy and Veilleux 2007; Luo 2011; Saha et al. 2010). Heat sensitivity has been shown to cause yield reduction in species of both temperate and tropical zones, but in general, tropical varieties often tolerate higher temperatures better, compared to varieties of the same crop species grown in temperate zones, as was shown for yard-long bean, cucumber and radish (Wahid et al. 2007; Momonoki and Momonoki 1993; Yamamoto et al. 2011). Similarly, warm-season annuals usually cope better with high temperatures than cool-season annuals. For example, for the warm-season annuals cowpea and rice, the maximum temperature for emergence is 37 and 40 °C, respectively (Yoshida et al. 1981; Akman 2009), while cool-season crops such as chickpea, lentils and lettuce show decreased germination rate at soil temperatures above 33, 24 and 32 °C, respectively (Covell et al. 1986; Hall 2001).

Various temperature thresholds of a range of crops, including cereals, horticultural and legume crops, have been reviewed in detail (Luo 2011). Most crops suffer if high temperatures are encountered during the vegetative growth period, as has been documented for both cool-season annuals like wheat (Porter and Gawith 1999) and *Brassica juncea* (Hayat et al. 2009) and for warm-season annuals, such as rice (Peng et al. 2004; Lyman et al. 2013), maize (Crafts-Brandner and Salvucci 2002), legumes (McDonald and Paulsen 1997) and tomato (Camejo et al. 2005).

However, for many crops, including rice, maize, soybean, legumes, rapeseed, sunflower and tomato, the reproductive stage appears to be even more vulnerable to temperature increase (Jagadish et al. 2014; Zinn et al. 2010; Barnabás et al. 2008; Hedhly et al. 2009). This is especially true during inflorescence/panicle development and during flowering, where heat may lead to flower abortion or reduced fertility, respectively (Maduraimuthu and Prasad 2014; Luo 2011).

### Heat and fertility

Reduced fertility is a common problem associated with heat, and has been found to be caused by high temperatures during meiosis and fertilization in various species, e.g., *Arabidopsis,* tomato, rice, cowpea and barley (Bac-Molenaar et al. 2015; Giorno et al. 2013; Jagadish et al. 2014; Ehlers and Hall 1998; Sakata and Higashitani 2008). The male reproductive organs and, in particular, pollen development are the most heat sensitive. Exposure to high temperature stress during flowering results in a reduction of viable and germinating pollen (Sato et al. 2006; Abiko et al. 2005; Prasad et al. 2006; Oshino et al. 2007; Jagadish et al. 2010; Zinn et al. 2010; Peet et al. 1998). For example, in rice, spikelet sterility occurs if temperatures exceed 35 °C for just 1 h (Yoshida et al. 1981; Endo et al. 2009). Tomato also has a dramatic decrease in fruit set in response to heat stress, especially when applied during microsporogenesis (Zhang and Yang 2014). During this stage, a short period at 40 °C or extended exposure just a few degrees above optimal temperature (32 °C rather than 26 °C during the day) results in male sterility (Sato et al. 2006; Giorno et al. 2013). Similarly, barley shows vulnerability to chronic mild heat stress (30 °C/25 °C day/night, for 5 days), by failure of tapetum differentiation and injuries to the microsporogenesis process (Sakata and Higashitani 2008).

Maternal tissues of the pistil and the female gametophyte have traditionally been considered to be more thermotolerant. However, malformations of the female tissues can occur in some species when subjected to heat. Embryo sac malformations have been reported in peach developed above 25 °C, in wheat at 30 °C and rapeseed at 32 °C, which consequently reduced the seed set in the latter two species (Hedhly 2011). In apricot, even a mild increase of 3 °C above control conditions during the last week of flower development resulted in shortening of the style and abnormal ovaries (Rodrigo and Herrero 2002). In addition to effects on male tissue, stigma receptivity is shortened by heat in cherry and peach, and ovule longevity is reduced in cherry and plum (Endo et al. 2009; Hedhly 2011). Such alterations result in a lack of synchrony between male and female reproductive tissues, ultimately leading to reduced fertilization efficiency. However, timely pollination does not guarantee fruit or seed set, as post-pollination processes such as pollen tube growth, fertilization, formation of the endosperm and embryo development were also shown to be heat sensitive (Peet et al. 1997; Erickson and Markhart 2002; Barnabás et al. 2008).

## Adaptation of cultivation methods to avoid heat stress

As part of the plant’s phenotype, yield is the result of the expression of the genotype (G), the environment (E) and their interaction (G × E). In the field of agriculture, management practices (M) are often included as a separate third factor, leading to the G × E × M model. Thus, yield improvement can in principle be achieved by adapting the genotype, as discussed later, the environment, or the management practices.

At the level of farming, a few technical and management adjustments may contribute to an increased ability of crops to cope with temperature changes. Firstly, assuming concomitant higher winter temperatures, the dates of planting can be adapted to avoid heat stress later in the growing season (Olesen and Bindi 2002; Easterling 1996; Rosenzweig and Tubiello 2007; Lotze-Campen and Schellnhuber 2009). Crop planting and harvesting dates from around the world have been recorded and used to make a so-called crop calendar. This calendar contains ~1300 planting and harvesting date observations for 19 crops, allowing estimation of the effects of planting time patterns at many geographic locations (Sacks et al. 2010). For example, in the US Midwest, early planting seems to be a successful strategy to avoid summer heat for maize and spring wheat (Reilly et al. 2003). Secondly, improvements in water management can alleviate heat stress in agriculture, as plants transpire to keep foliage temperature under control. One option is shifting from rain-fed to irrigated agriculture, including low-cost “rainwater harvesting” practices. Additionally, adjusting the timing of irrigation may ensure a crop’s water supply at critical, temperature-sensitive stages (Easterling 1996; Smithers and Blay-Palmer 2001; Smit and Skinner 2002; Lotze-Campen and Schellnhuber 2009).

Another strategy of avoiding heat stress is to change the “environment” factor of the GxExM model, by shifting the geographical location of crop cultivation. Although this strategy is drastic, it is already occurring, for instance, in Australia’s wine industry, where several large wine producers have bought new properties in cooler regions to maintain vineyards in the future (Chapman et al. 2012; Park et al. 2012). It is also occurring for other crops, like maize and rice (Kenny et al. 1993; Duzheng 2003; Tchebakova et al. 2011).

While adapting to climatic changes via alterations in cultivation may be possible for some crops, heat stress cannot be avoided by this approach alone (Reilly et al. 2003; Tubiello et al. 2002). For example, in areas where water is a scarce and valuable resource, improving water supply might not be an option. Similarly, moving cultivation areas geographically might be a solution for Australia’s wine industry, but is not for Australia’s wheat farmers, because winter temperatures at lower latitudes near the sea are also too high (Chapman et al. 2012). Because of these limitations, the introduction of more heat-tolerant cultivars or shifting to other crops is essential to maintain food production in areas with increasing temperatures.

## Conventional breeding for heat tolerance

Increasing temperature tolerance by conventional breeding is an obvious approach to reduce the negative effects of heat on crop yield. Usually, breeding programs are carried out in a climacteric region similar to that where the crop will be produced eventually. Thus, the selection of breeding lines for relatively hot regions takes place under hot conditions (Mickelbart et al. 2015). This implies that in hot regions, thermotolerance traits are “passively” selected for by locally operating breeders. Considering that cultivars from warmer regions are often more heat-tolerant than those from cooler regions, it seems that this technique has been rewarding (Tonsor et al. 2008; Smillie and Nott 1979; Yamamoto et al. 2011; Momonoki and Momonoki 1993; Kugblenu et al. 2013). Conventional breeding has also been used to intentionally develop new heat-tolerant crop genotypes. For example, a variety of broccoli has an improved head quality thanks to early maturation, because this trait prevents hot days later in season to affect the heat-sensitive flower initiation developmental stage (Farnham and Bjorkman 2011). In addition, new varieties of cowpea showed higher average grain yield when grown under hot and long days during reproduction (Ehlers and Hall, 1998) and recurrent selection has also been successful for improving wheat yield using ancestor *T. tauschii* as a gene donor, leading to increased rates of grain filling and larger grains in BC1F6 plants (Gororo et al. 2002). Finally, in potato breeding a genetic gain was obtained after three cycles of recurrent selection for heat tolerance leading to strong increase in yield up to 37.8 % (Benites and Pinto 2011).

Although conventional “yield” breeding has succeeded in developing heat-tolerant lines, the ultimate genetic and physiological bases of the improvements remain unclear. This prevents the development of molecular or other biomarkers, which would assist germplasm screening for improved heat tolerance and allow for efficient breeding of the complex trait. Another drawback of conventional breeding is that the programs are often based on crossing relatively advanced starting material, which has already been used in the particular breeding areas specifically related to the market segment that is targeted. This implies that the potential gain in heat tolerance level is limited by the low genetic diversity (Ladizinsky 1985; Paran and Van Der Knaap 2007).

## Advanced breeding for heat tolerance

### Intra-specific QTL discovery

Heat tolerance seems to be polygenic, which might explain why the genetic basis of heat stress tolerance in plants is poorly understood (Wahid et al. 2007; Ainsworth and Ort 2010; Collins et al. 2008). In order to improve knowledge about thermotolerance at the genetic level, many efforts have been made to identify quantitative trait loci (QTL) in segregating mapping populations. Jha et al. (2014) recently listed QTLs associated with heat tolerance in various plants, including *Arabidopsis*, azuki bean, barley, brassica, cowpea, maize, potato, rice, sorghum, tomato and wheat. In this paper, the authors showed several types of genetic markers linked to different traits of interest which spanned the various aspects of a plant’s vulnerability to heat. This included QTLs for yield traits, such as fruit set or grain filling rate, under heat. Also, QTLs for several heat tolerance-related traits have been discovered, such as for lower canopy temperature during vegetative and reproductive stages and higher chlorophyll fluorescence in wheat (Pinto et al. 2010; Lopes et al. 2013; Vijayalakshmi et al. 2010). High chlorophyll fluorescence represents heat-tolerant photosynthesis, and lower canopy temperature reflects efficient water uptake which has been associated with deep rooting (Pinto and Reynolds 2015). A major QTL for high-temperature seed germination capacity in lettuce, *Htg6.1,* colocates with a temperature-sensitive gene encoding an abscisic acid biosynthesis enzyme (*LsNCED4)* (Argyris et al. 2008, 2011). In potato, nine QTLs for internal heat necrosis in tubers were detected that each explain between 4.5 and 29.4 % of the phenotypic variation (McCord et al. 2011). Many studies have focused on the effect of high temperature on reproductive characteristics, including pollen germinability, pollen tube growth, grain weight, days to heading, grain filling and post-anthesis leaf senescence, fruit set and quality traits such as white-back kernels in rice. In maize, five and six QTLs for pollen quality and tube growth have been identified with a high heritability of 0.64 and 0.68, respectively. However, the pollen tests were performed in vitro and might not be representative of the situation in vivo (Frova and Sari-Gorla 1994). Lastly, in tomato, six QTLs were identified that explain 33 % of the phenotypic variation related to fruit set at high temperature (Ventura et al. 2007). A recent QTL study in rice focussed on spikelet fertility under high temperature (Ye et al. 2015). This study followed up previous work (Ye et al. 2012) and confirmed the presence of a recessive QTL on chromosome 4 which contributes 15 % higher rice spikelet fertility under heat stress compared to plants without the QTL (Ye et al. 2015). Heat tolerance QTLs on this chromosome have been identified in different populations of heat-tolerant rice varieties (Ye et al. 2012, 2015; Xiao et al. 2010). Ye et al. (2015) showed that the QTL is located in a highly conserved chromosomal region. Unfortunately, this limits mapping resolution and causal gene identification. More advanced approaches such as the use of multi-parent advanced generation inter-cross (MAGIC) populations were suggested as a way to introduce more genetic variation and determine the genes involved in thermotolerance of spikelet fertility (Ye et al. 2015).

Besides dedicated mapping populations, QTLs can be detected via exploration of natural populations. As noted previously, linkage mapping can be considered as being useful for identification of major genes and QTLs. However, due to the limited number of generations and thus recombination events, those QTLs cover a relatively large region and gene identification requires time-consuming fine-mapping processes. Exploiting natural diversity panels avoids these hurdles. Using a genome-wide association approach, the linkage decay is fast, therefore providing a much higher resolution. Consequently, fine mapping is often not necessary for identification of candidate genes (Bergelson and Roux 2010). So far, genome-wide association study (GWAS) panels have been established in *Arabidopsis* and several crops including maize, rice, sorghum and foxtail millet (Buckler et al. 2009; Huang et al. 2011; Jia et al. 2013; Morris et al. 2013). Maize and rice seem to be the two major models for crop GWAS, considering the magnitude of resources already developed and published for these species (Huang and Han 2014). So far, QTLs explaining transition of the vegetative to generative stage have been established in these crops (Buckler et al. 2009; Huang et al. 2011). Although those QTLs are not related to heat tolerance directly, QTLs explaining flowering time are of interest for heat tolerance breeding, as early flowering might enable a plant to complete the heat-sensitive reproductive processes before late-season heat episodes (Ishimaru et al. 2010). QTLs directly associated with high-temperature-induced reduction in fertility have recently been revealed in *Arabidopsis*. An *Arabidopsis* inflorescence can have flowers at many different developmental stages, and by measurement of the plant silique length after short-term heat stress, sensitivity of the different reproductive stages was determined. Meiosis, fertilization and early embryogenesis were shown to be most vulnerable to heat. GWAS study of this experimental setup revealed four QTLs related to specific developmental stages. Three QTLs were responsible for sensitivity of pre-anthesis reproductive processes including male and female meiosis, while one QTL explained population variation of early embryogenesis heat sensitivity. A strong negative correlation between flowering time and silique length was detected, which were strongly and moderately associated with the same SNP, respectively. Interestingly, this SNP has been linked to the flowering time repressor *FLC*, suggesting a role for the regulation of flowering time in the heat stress response (Bac-Molenaar et al. 2015). As *Arabidopsis* is a member of the *Brassicaceae* family, the results might provide insights for breeding within this family.

Altogether, the QTL studies in different crops all identified multiple QTLs per trait, varying from two in rice and azuki bean (enhancing spikelet and pollen viability under heat stress, respectively) up to 34 in barley for several heat-related traits (e.g., number of spikes per plant and days until heading). This demonstrates that heat tolerance is dependent on a range of factors and QTLs which seem to vary between crops (reviewed in Jha et al. 2014).

### Expanding genetic diversity with crop-related wild species

Crop domestication may be regarded as the first stage of plant breeding, resulting in dramatic morphological and physiological modifications to meet human needs, including seed and fruit size and number, seed shattering, seed dormancy, photoperiod and flowering time, taste, nutrition and overall plant architecture (Meyer and Purugganan 2013; Gross and Olsen 2010). Domestication inevitably involves a genetic bottleneck due to selection and breeding of similar lines with favorable traits, after which only a subset of the genes and alleles available in the wild progenitor gene pool are present among crop cultivars (Godfray et al. 2010; Ladizinsky 1985; Olsen and Wendel 2013). This reduction in diversity seems to have led to a loss of abiotic stress tolerance traits, since many wild relatives and landraces are more tolerant to stresses compared to domesticated crops (Dolferus 2014; Maduraimuthu and Prasad 2014). Therefore, the identification of superior wild alleles that are lacking in cultivated germplasm has become of great interest (Tanksley and McCouch 1997; Grandillo et al. 2007; Lippman et al. 2007; Feuillet et al. 2008). For example, introgression of wild alleles has resulted in crop improvement in several cereals such as rice (Atwell et al. 2014), wheat (Pradhan et al. 2012) and maize (Prasanna 2012). However, exploiting wild relatives as a source of novel alleles has been hindered by the introduction of linked, undesirable traits, compounded by a lack of molecular markers for precision breeding. (Dolferus 2014). Only recently, new sequencing methods have made it cost-effective to re-sequence complete genomes, as has been done for wild tomato (Aflitos et al. 2014), cucumber (Qi et al. 2013), sorghum (Mace et al. 2013), grape (Lijavetzky et al. 2007), soybean (Li et al. 2013), rice (Xu et al. 2010) and maize (www.panzea.org). Difficulties still arise when QTLs are crossed in a particular genetic background and show a smaller or no effect at all. Due to the potential for unfavorable epistatic interactions, it is difficult or even impossible to predict in advance whether a QTL might be transferable to elite backgrounds (Podlich et al. 2004; Collins et al. 2008).

Despite difficulties associated with fine mapping, identification of the causal gene was successful for a major QTL for seedling survival rates under high temperature in African rice (*Oryza glaberrima*) (Li et al. 2015). This species has several advantageous traits, including tolerance to drought, salinity and heat (Sakai et al. 2011). The heat tolerance QTL was associated with a single gene, *Thermo*-*tolerance 1* (*TT1*), coding for an α2 subunit of the 26S proteasome which is involved in the degradation of ubiquitinated proteins. This gene is thought to protect cells from heat stress by enhancing efficient elimination of cytotoxic denatured proteins and maintaining heat-response processes (Li et al. 2015). Comparison of the sequence of *TT1* from the Asian and African cultivated parental lines, *Oryza sativa* ssp. *japonica* and *Oryza glaberrima*, revealed three exonic SNPs, one of which resulted in an amino acid substitution. Interestingly, *TT1* was suggested to be a major determinant for variation in thermotolerance among *O. sativa* varieties: geographical distribution of three *TT1* haplotypes showed that environmental pressure was responsible for the selection of the *TT1* heat tolerance locus. In addition to a higher seedling survival, near-isogenic lines (NILs) containing the *O. glaberrima TT1* allele showed a higher thermotolerance at flowering and filling stages compared to NILs with an *O. sativaTT1* allele. Overexpression of this gene not only led to enhanced seedling thermotolerance in other rice species, but also in *Arabidopsis* and *Festuca elata* (Li et al. 2015), showing the enormous potential of this allele to enhance crop productivity under high-temperature stresses.

Together, these studies indicate that QTL analysis and subsequent fine mapping and cloning are promising ways to identify loci and genes for heat tolerance. Several candidate genes have been proposed, but characterizing the causal gene underlying a heat tolerance QTL remains challenging. However, for breeding purposes, the exact underlying genes do not have to be known. Using molecular markers based on linked flanking polymorphisms of a QTL, a QTL can still be successfully introduced into crossable breeding germplasm.

## Discovery of thermotolerance genes

In response to high-temperature stress, plants modulate the expression of a plethora of genes. These genes and their annotation could help to identify the processes that are induced or repressed such as those involved in acclimation and protection to heat stress.

Transcriptional profiling has been performed during the onset and recovery of heat stress, between stressed and unstressed plants, or between heat-tolerant and heat susceptible variants. Such analyses have been performed in many crops, e.g., rice, tomato, barley, brassica and grape (Frank et al. 2009; Sarkar et al. 2014; Frey et al. 2015; Bita et al. 2011; Liu et al. 2012; Mangelsen et al. 2011; Dong et al. 2015). An extensive summary of recent transcriptomic analysis in plant species was published by Lavania et al. (2015). This summary revealed that plants reprogram their signal transduction pathway, transcription factors and proteins associated with metabolism in a conserved manner. Although the studies were performed in different crops which were exposed to different heat regimes, there was considerable similarity in the heat stress-responsive genes.

For example, there is a conserved induction of genes encoding for enzymes that govern the fluidity of membranes upon heat stress. In agreement with this, overexpression of one of the enzymes: *glycerol*-*3*-*phosphate acyltransferase,* resulted in increased saturation of the thylakoid membrane lipids of transgenic tobacco plants, showed a faster recovery after heat stress compared to wild-type plants (Yan et al. 2008). When plants are exposed to heat, reactive oxygen species (ROS) are formed as a by-product in various aerobic metabolic pathways in different cellular compartments (Miller et al. 2009; Wang et al. 2014a; Chou et al. 2012; Dat et al. 1998; Volkov et al. 2006; Wu et al. 2012; Vacca et al. 2004; Mostofa et al. 2013), and cause cellular damage to membranes, proteins, lipids and DNA (Volkov et al. 2006; Wu et al. 2012; Bokszczanin et al. 2013; Baker and Orlandi 1995; Giardi et al. 1997; O’Kane et al. 1996; Larkindale and Knight 2002). In order to prevent damage to the cell and regain redox homeostasis, a typical response to heat is hyper-activation of the ROS scavenging machinery. The transcript and protein level of genes responsible for ROS scavenging are increased under heat stress in many different plant species (Chou et al. 2012; Chao et al. 2009; Mittal et al. 2012; Suzuki et al. 2013) and this has been associated with basal heat tolerance (Almeselmani et al. 2006; Bhattacharjee 2012; Gupta et al. 1993; Kang et al. 2009; Rui et al. 1990; Wang et al. 2014b; Sairam et al. 2000; Badiani et al. 1993). A ROS scavenging-related gene that seems to be important for thermotolerance is glutaredoxin (GRX). This small ubiquitous protein is a regulator in diverse cellular processes and oxidative stress response, and its function is conserved in prokaryotes and eukaryotes (Lillig et al. 2008; Cheng et al. 2009; Wu et al. 2012). As a critical component of ROS metabolism, *Arabidopsis AtGRXS17* may be crucial for temperature-dependent postembryonic growth and development (Cheng et al. 2011). Indeed, improvement of plant heat stress tolerance has been achieved by increasing antioxidant enzyme and GRX activities (Almeselmani et al. 2006; Badiani et al. 1993; Gupta et al. 1993; Rui et al. 1990; Sairam et al. 2000; Wu et al. 2012; Chen et al. 2013). Probably, the best-studied mechanism in response to heat stress is the production of heat shock proteins (HSPs) upon exposure to high temperature (Wang et al. 2004). By acting as molecular chaperones, HSPs prevent deleterious protein conformations and eliminate non-native aggregations formed during stress (Morimoto 1998; Boston et al. 1996; Vierling 1991). Strong transcriptional up-regulation of a number of HSPs by heat stress has been shown in plants and many other organisms. The expression of HSPs and various other heat-responsive genes is controlled by heat shock transcription factors (HSFs) (Kotak et al. 2007). Experiments in *Arabidopsis,* rice, tobacco and tomato have shown that enhanced thermotolerance can be gained by overexpressing HSPs or HSFs (reviewed in Grover et al. 2013).

## Conclusion

Despite the urgent need to improve crop heat tolerance, a very limited number of heat-tolerant varieties have been developed. The development of new varieties through plant breeding is expensive and time-consuming (Lotze-Campen and Schellnhuber 2009; Rosenzweig and Tubiello 2007)—for annual crops, it may take 10–30 years to introduce specific adaptations (Chapman et al. 2012; Rosenzweig and Tubiello 2007; Smit and Skinner 2002; Olesen and Bindi 2002). Therefore, it is very important that genetic variation for the trait can be identified and characterized efficiently in order to introduce it in a breeding program. At this point, fundamental research plays an important role as knowledge on molecular physiology of the plant heat response can speed up the cloning of causal genes after QTL identification. Furthermore, fundamental knowledge may be used to generate leads for biotechnological modification of heat tolerance traits. Although genetic modification is controversial in some parts of the world, the products generated with new gene editing techniques may be in the near future classified as non-transgenic in the EU (https://www.euroseeds.eu/new-plant-breeding-techniques). Gene editing techniques involve several site-directed nuclease techniques such as SDN-1 and SDN-2 using zinc-finger nucleases (ZFNs), transcription activator-like effector nucleases (TALENs), meganucleases and the clustered regularly interspaced short palindromic repeats (CRISPR)/CRISPR-associated Protein9 (Cas9) system (CRISPR/CAS9) (Sander and Joung 2014; Lusser et al. 2012; Hartung and Schiemann 2014; Mahfouz et al. 2014). Several heat-tolerant transgenic plant species have been generated already. However, a major finding of fundamental research is that the plant heat stress response is highly complex, with challenges that may be tissue, developmental stage and even species specific (Hedhly 2011; Maduraimuthu and Prasad 2014). Thus, heat tolerance should not be regarded as a single trait, and as such, it is unlikely that a general strategy can be developed to generate heat tolerance. For the near future, it will be important to evaluate to what extend the current research data obtained from model species such as *Arabidopsis* is translatable to crop species. Fortunately, genomes of most of the important crop species have been sequenced and annotated, making it possible to transfer technologically advanced methods to the crop species themselves. A remaining limitation is the space and expertise necessary to grow a crop under controlled, representative conditions and geographic location. Herein lies an opportunity for academic research groups to closely work together with breeding companies so that each can benefit from the other’s expertise.

Despite widely being regarded as essential, academic research is expensive and companies are often unwilling or unable to subsidize research when no short-term payback is foreseen. At the same time, research with a high certainty of application may not be suitable or challenging for academics, many who strive to publish in higher-ranked scientific journals. A solution to this problem may lie in tripartite collaboration between academia, the private sector and governments, based on the shared aim of contributing to sustainable food production for a growing population in a warming world. Well-balanced investments may have synergistic effects on academic research output and the potential for application of findings.

In conclusion, in order to achieve success, combined efforts of plant physiologist, molecular biologists and crop breeders are required. Given the importance of global food security, the need for a versatile and linked global strategy and multidisciplinary collaboration involving governmental agencies, companies and academics is particularly evident.

### **Author contribution statement**

N.D., W.V. and I.R. wrote the manuscript. All authors read and approved the manuscript.
